# Medical Management of Septic Arthritis of the Sternoclavicular Joint With Extended-Spectrum Beta-Lactamase-Producing Escherichia coli: A Case Report

**DOI:** 10.7759/cureus.23969

**Published:** 2022-04-09

**Authors:** Sara Alhariri, M Ammar Kalas, Mariam Hassan, Jordan T Carter, S Reshad Ghafouri, Fatma Dihowm

**Affiliations:** 1 Internal Medicine, Texas Tech University Health Sciences Center El Paso, El Paso, USA; 2 Orthopaedics, University of Texas Health Science Center at San Antonio, San Antonio, USA

**Keywords:** rheumatology, ultrasound (us), septic arthritis, sternoclavicular joint (scj), extended spectrum-beta lactamase (esbl)

## Abstract

The sternoclavicular joint (SCJ) is an uncommon location for septic arthritis to occur in. Due to the rarity of the condition and the nonspecific symptoms, SCJ septic arthritis can be missed or mislabeled as osteoarthritis or muscle strain. Accurate history and physical examination is crucial for recognizing this condition. With the potential life-threatening complications that may ensue, SCJ septic arthritis has traditionally been managed surgically. This ranges from simple incision and drainage to resection of the joint. However, in cases where there is not enough fluid for incision and drainage, a trial of medical management with antibiotics can be attempted. We herein describe a case of a 58-year-old male who presented with nonspecific anterior chest wall and neck pain. Chest X-ray and ultrasound of the anterior chest wall was normal; however, magnetic resonance imaging (MRI) of the chest showed a small effusion without other complications. His blood cultures grew extended-spectrum beta-lactamase (ESBL)-producing *Escherichia coli,* rendering this as his hematogenous source of septic arthritis. The ESBL was from a left-sided obstructing kidney stone that resulted in pyelonephritis, which was confirmed via computed tomography of the abdomen. His effusion was too minimal to drain; therefore, he was managed medically with intravenous (IV) antibiotics along with a left ureteral stent placement, and he had a full recovery. This case represents the ability for SCJ septic arthritis to be managed medically with IV antibiotics, especially when the diagnosis is caught early without complications. The role of MRI is indispensable for coming to the diagnosis, as it is capable of detecting complications that ultimately dictate management. Additionally, this case highlights the unique microorganism, ESBL-producing *E. coli* causing the SCJ septic arthritis*, *a finding that has been rarely reported in the literature as the majority of microorganisms that have been previously documented are either *Staphylococcus aureus *or *Pseudomonas aeruginosa.*

## Introduction

Septic arthritis of the sternoclavicular joint (SCJ) is a rare, but potentially deadly disease accounting for less than 1% of all joint infections [1]. As with other amphiarthrodial joints, infection is typically diagnosed in middle-aged patients with a mean age of 45 years. It is associated with intravenous (IV) drug use, systemic diseases (e.g. diabetes mellitus), and distant infections with hematogenous spread [2]. Early diagnosis is crucial as complications such as osteomyelitis, mediastinitis, and rapidly progressive necrotizing myositis can occur and carry a high morbidity and mortality [2,3].

Due to the rarity of the condition, there have been no large-scale studies conducted to evaluate the optimal diagnosis and treatment modalities, with most of the data being derived from case reports and case series. 

## Case presentation

A 58-year-old male presented to the emergency room with a three-day history of continuous anterior chest wall and neck pain radiating to the left shoulder, rated as 10 out of 10 in severity. Pain was described as sharp in quality and he tried ibuprofen without relief of the pain. He had a past medical history of hypertension and was taking amlodipine 10 mg and hydrochlorothiazide 25 mg daily. He had subjective fever, chills, nausea, vomiting, and left-sided flank pain. He denied any history of trauma to the neck, or any other joint pain. He was an active smoker and smoked five cigarettes a day, and drank alcohol socially, and did not participate in recreational drug use.

Upon presentation, his temperature was 36.3°C, blood pressure was 137/79 mmHg, heart rate was 91 beats per minute, and respiratory rate was 18 breaths/minute, with an oxygen saturation of 96% on room air. Physical examination revealed warmth, erythema, and tenderness to palpation of the left sternoclavicular joint without notable swelling or effusion. The range of motion of his left glenohumeral joint was normal. He had left costovertebral angle tenderness without any abdominal tenderness.

The patient had an acute kidney injury with a creatinine level of 1.7 mg/dL that resolved with IV hydration (Table 1). His urine drug screen was negative. The patient was initially started on IV cefepime for gram-negative coverage given a high suspicion for intra-abdominal source of infection. The urine culture grew extended-spectrum beta-lactamase (ESBL)-producing *Escherichia coli*. Two out of two blood cultures grew ESBL-producing *E. coli, *with the sensitivities similar to that of the urine culture. The cultures were sensitive to ertapenem, and he was switched from IV cefepime to IV ertapenem.

**Table 1:** Initial lab work-up on presentation ESR, erythrocyte sedimentation rate; CRP, C-reactive protein; ALT, alanine transaminase; AST, aspartate transaminase; ALP, alkaline phosphatase; MCV, mean corpuscular volume; RBC, red blood cell; WBC, white blood cell; BUN, blood urea nitrogen

Laboratory investigations	
Complete blood cell count with differentials	
WBC	27.1 x 10^3^/UL
Hemoglobin	15.4 g/dL
MCV	89.4
Platelet	170 x 10^3^ U/L
Neutrophil absolute	22.83 x 10^3^ U/L
Bands absolute	0.95 x 10^3^ U/L
Bands percent	3.5
Myelocyte absolute	0.49 x 10^3^ U/L
Lymphocyte absolute	2.14 x 10^3^ U/L
Monocyte absolute	0.71 x 10^3^ U/L
Complete metabolic panel	
Sodium	133 mmol/L
Potassium	4.9 mmol/L
Chloride	109 mmol/L
Bicarbonate	19 mmol/L
BUN	50 mg/dL
Creatinine	1.7 mg/dL
Calcium	8.2 mg/dL
Albumin	2.4 g/dL
AST	17 IU/L
ALT	33 IU/L
ALP	130 IU/L
Others	
ESR	25 mm/hr
CRP	16.1 mg/dL
Procalcitonin	3.9
Lactic acid	0.9 mmol/L
Troponin	<0.012 ng/mL
Urinalysis	
Glucose	Negative
Bilirubin	Negative
Ketone	Negative
Blood	Moderate
Protein	Negative
pH	5
Leukocyte esterase	Moderate
Nitrite	Positive
Urine microscopy	
WBC	>50
RBC	11-20
Bacteria	Moderate
Squamous epithelial	Negative
Uric acid crystals	Occasional

**Figure 1:**
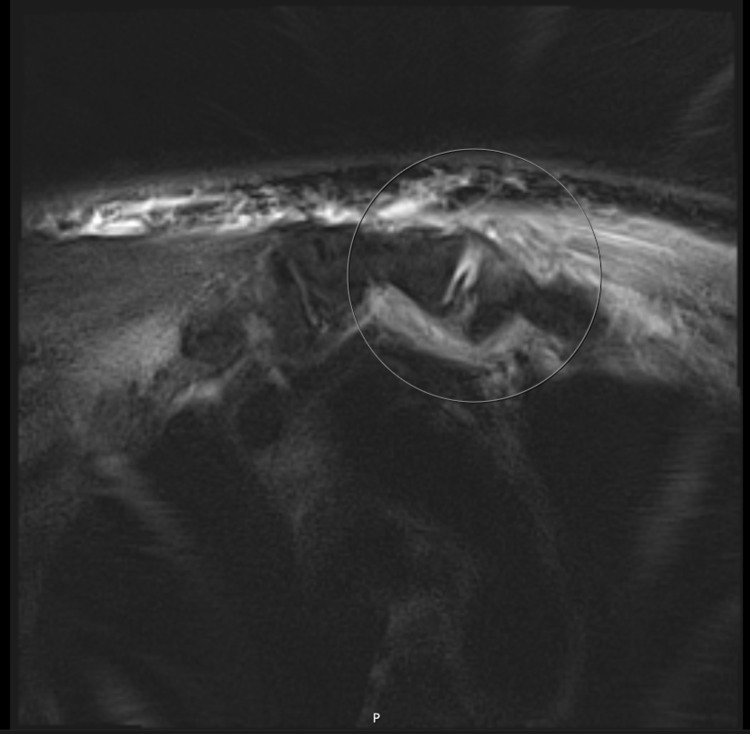
Magnetic resonance imaging of the chest showing asymmetric effusion of the left sternoclavicular joint with soft tissue edema (white circle)

Initial diagnostic imaging included a chest X-ray (CXR) and ultrasound (US) of the anterior chest, both of which were unremarkable. Even though the US did not reveal an effusion, the orthopedic team was consulted and recommended magnetic resonance imaging (MRI) of the chest for better delineation of possible septic arthritis. MRI chest revealed a small effusion of the left SCJ, findings consistent with early septic arthritis (Figure 1). Suspecting an abdominal source of his ESBL-producing* E. coli*, computed tomography (CT) of the abdomen was ordered and demonstrated a left-sided 5-mm obstructing kidney stone in the mid-left ureter with associated ureteritis and left pyelonephritis. Urology was consulted and he underwent a left ureteral stent placement.

The left-sided SCJ effusion was too minimal to be drained by interventional radiology. As a result, fluid analysis and culture from the SCJ could not be obtained. It was therefore recommended by the infectious disease team to use the blood culture as guidance to treat the underlying septic arthritis, as his bacteremia led to seeding of the SCJ. This bacteremia source was from the kidney stone that led to ureteritis and pyelonephritis. The patient received medical management with IV ertapenem for his ESBL for a total of three weeks post-stent placement.

He was discharged with home health care for IV antibiotic management, and the follow-up one month later in the infectious disease clinic indicated that he no longer had chest wall pain. His repeat blood cultures were negative, his white blood cell count went down to normal at 6 x 10^3^/L, and his C-reactive protein level went down to 1 mg/dL. He responded well to the medical therapy and a chest US scan obtained revealed no evidence of effusion.

## Discussion

Septic arthritis of the small joints such as the acromioclavicular joint is relatively rare with an incidence of approximately 8/100,000 people [4]. However, SCJ septic arthritis is extremely rare and seen in less than 1% of the general population [1]. Its prevalence is estimated at 7% in a 10-year period [5]. For unclear reasons, septic arthritis of the SCJ tends to occur in younger patients with a median age of 45 years, versus septic arthritis of other joints wherein the median age is 65 [2]. This could be explained by the fact that this joint can be sclerosed in the elderly, or that there is a higher risk of IV drug abuse in the younger population.

SCJ septic arthritis is more commonly seen in males, diabetics, renal failure patients, those on immunosuppressive therapy, or those with an indwelling central venous catheter [6]. Other uncommon predisposing risk factors include post-tracheostomy, infected pacemaker, or post-periarticular steroid injection in the shoulder [3,7,8]. In a study, the presence of a distant site infection was found to be the second most important risk factor when reviewing 180 patients with septic arthritis of the SCJ [2]. Examples of such distant site infections included pneumonia, cellulitis, endocarditis, and tuberculosis, with one patient having urosepsis (which our patient also had). Additionally, the biggest risk factor was injection drug use, and septic arthritis of the SCJ accounts for 17% of septic arthritis cases in drug abusers [9]. Yet it can also be seen even in patients without any identifiable risk factors [10].

The SCJ is a saddle-type synovial joint connecting the manubrium of the sternum with the clavicle and the first costal cartilage. It has tough anterior and posterior ligaments rendering it immobile [1]. It also has a fibrous capsule that divides the joint into two synovial spaces [11]. The SCJ is believed to be vulnerable to bacterial seeding due to its proximity to the subclavian vein. It is also believed to be vulnerable to traumatic injuries secondary to its superficial location on the anterior chest wall [12].

Presenting symptoms may include fever (65%), chest pain (78%), neck pain (2%), with up to only 4% of patients presenting with swelling of the SCJ [2]. While it typically presents unilaterally in 95% of cases, there are case reports of it presenting bilaterally [6]. The median duration of symptoms is approximately 14 days [2]. Abscess formation can be commonly seen at the time of diagnosis [13]. The location of the abscess varies and can include chest wall abscess, or rarely cervical abscess [14,15]. Because of the subtle symptoms, diagnosis may be delayed due to the attribution of clinical symptoms to other diagnoses such as muscle strain or osteoarthritis.

Laboratory findings include leukocytosis in up to 56% of patients, with bacteremia in up to 62% [2]. SCJ septic arthritis often yields positive blood culture, especially in comparison to non-SCJ septic arthritis [13]. The most commonly isolated organism in this condition is *Staphylococcus aureus *(49%) followed by *Pseudomonas aeruginosa *[2]. In a review of 180 cases with SCJ septic arthritis, only 5% of all cases were caused by *E. coli* with no documented cases of ESBL-producing organisms [2]. Other rare organisms include *Streptococcus dysgalactiae *and *Clostridium perfringens* [16,17].

Apart from a thorough physical examination with an emphasis on the SCJ in conjugation with laboratory diagnostics, imaging can aid in diagnosis. CXR is typically ordered as the first imaging modality whenever a patient presents with chest pain; a retrospective study revealed that 10 out of 13 patients with septic arthritis of SCJ had a normal CXR [1]. The abnormalities in the other three patients included joint space widening, presence of a soft tissue shadow, and evidence of osteolysis in the clavicle [1]. It can take up to 12 days for a CXR to reveal soft tissue swelling [18]. Our patient's first imaging was a CXR and was negative for any acute findings.

A retrospective study of 10 patients with SCJ septic arthritis revealed that 2 to 11 radiological tests were needed to establish the diagnosis, and that CT or MRI was needed in all patients [18]. MRI as the initial diagnostic test had the best sensitivity, and can show changes within one to two days with no diagnostic delay. Using CT led to a 1.5-week diagnostic delay and using the US had a lower sensitivity rate, making it difficult to differentiate between infectious causes and rheumatological inflammation. CT findings include abscess, air, and osteonecrosis [19]. However, in clinical practice, typically MRI is not ordered as the initial diagnostic modality unless suspicion of the condition is high.

Traditionally, septic arthritis of the SCJ has been managed surgically. This ranges from simple incision and drainage to extensive debridement and reconstruction. During reconstruction, the medial third of the clavicle is incised along with the manubrium, with an en bloc resection. Subsequently, an ipsilateral pectoralis major muscle flap would be advanced. A wound vacuum dressing would then need to be placed to facilitate granulation tissue formation. However, these surgical procedures do not come without complications. One retrospective study analyzed 44 patients who underwent muscle flaps after SCJ resection. Rib resection in combination with the SCJ led to an increased need for serial debridements, an increase in eight days from debridement to reconstruction, and a longer hospital stay [19]. In Song's retrospective study of seven patients, five out six patients who had an incision and drainage had failure of infection resolution and all seven patients who had aggressive surgical intervention were cured [20].

More recently, medical management alone was found to be successful even in the presence of clavicular osteomyelitis. One study comparing SCJ septic arthritis to non-SCJ septic arthritis revealed that from 33 patients diagnosed with SCJ septic arthritis, only 11 were treated surgically [13]. The typical duration of antibiotic treatment ranges from four weeks in uncomplicated cases to six weeks if there are complications such as osteomyelitis [2]. Because *S. aureus *is the most common organism, empiric coverage should include gram-positive coverage such as cefazolin, or vancomycin if methicillin-resistant *S. aureus* is suspected. Because our patient had ESBL-producing *E. coli* bacteremia, our antibiotic regimen targeted this multidrug-resistant organism with ertapenem. Antibiotic choice, in this case, was guided by blood cultures due to the lack of appreciable fluid on US, as is common in the literature.

If left untreated, complications can occur including but not limited to mediastinitis, abscess formation, osteomyelitis, empyema, and even death [5]. Complications are thought to be secondary to lymphatic spread or capsular rupture [2].

## Conclusions

Our patient had an identifiable source of infection that was his left-sided pyelonephritis secondary to nephrolithiasis. His urosepsis with ESBL-producing *E. coli *bacteremia led to the hematogenous spread to the left sternoclavicular joint, ultimately resulting in septic arthritis. Our patient represents a unique case in the literature due to his infection with ESBL-producing *E. coli *and a successful outcome with nonsurgical management. The case also highlights the indispensable role MRI carries in those with early stages of septic arthritis of the SCJ, as repeated US was unrevealing of any fluid.
